# Bisphosphonate-Related Atypical Femoral Fractures: A Comprehensive Review

**DOI:** 10.7759/cureus.96208

**Published:** 2025-11-06

**Authors:** Ahmed Mohamed, Usman Fuad, Mohamed Abdelazim, Alaa Elasad, Prabhakar Bhamidipati

**Affiliations:** 1 Trauma and Orthopaedics, Royal Cornwall Hospital, Truro, GBR; 2 Internal Medicine, Royal Cornwall Hospital, Truro, GBR; 3 General Practice, Zagazig University, Zagazig, EGY

**Keywords:** bisphosphonate-related fracture, bone turnover markers, drug holiday, incomplete atypical femoral fracture, transverse fracture pattern

## Abstract

Bisphosphonates have become a cornerstone in the management of osteoporosis and other bone-related conditions over the past few decades. While these medications have proven highly effective in reducing the risk of typical osteoporotic fractures, a concerning paradox has emerged: long-term use may be associated with a rare but serious complication known as atypical femoral fractures (AFFs). These unusual breaks occur in the shaft of the femur and differ significantly from common osteoporotic fractures in terms of location, appearance, and mechanism.

This review explores the nature of these fractures by examining their characteristics, underlying mechanisms, risk factors, clinical presentation, diagnostic approaches, and management strategies. Understanding this complication is essential for clinicians who prescribe bisphosphonates and for patients who take them, as it highlights the importance of balancing the substantial benefits of these medications with their potential risks - particularly with prolonged use.

## Introduction and background

Osteoporosis is one of the most significant public health challenges in aging populations worldwide [1,2]. This condition weakens bones, making them fragile and more likely to break. The introduction of bisphosphonates has revolutionized osteoporosis treatment, as they help maintain bone density and prevent fragility fractures of the hip, spine, and wrist [3].

Bisphosphonates work by slowing down the natural process of bone breakdown, allowing bone-building processes to catch up and strengthening the skeleton [4,5]. The most commonly used medications in this class include alendronate, risedronate, ibandronate, and zoledronic acid. These drugs have been used by millions of patients and have demonstrated remarkable success in reducing fracture risk. Studies have shown reductions of up to 50% for hip fractures and even greater reductions for spinal fractures [6-10].

However, around 2005, unexpected phenomena began to appear in the medical literature. Clinicians reported unusual femur fractures in patients who had been taking bisphosphonates for several years [11]. These fractures differed from typical osteoporotic breaks (Table 1). They occurred in the shaft of the femur rather than at the hip joint, often with minimal or no trauma, and showed characteristic patterns on X-rays. Some patients even experienced warning symptoms for weeks or months before the bone broke [12,13].

**Table 1:** Comparison of Typical vs. Atypical Femoral Fractures

Feature	Typical Osteoporotic Fracture	Atypical Femoral Fracture
Location	Femoral neck/intertrochanteric	Femoral shaft (subtrochanteric/diaphyseal)
Trauma	Low-energy fall	Minimal or no trauma
Fracture pattern	Spiral, oblique, comminuted	Transverse or short oblique
Prodromal symptoms	Rare	Common (weeks to months of pain)
Bilateral occurrence	Rare	20-30%
X-ray findings	Typical osteoporotic changes	Cortical thickening, medial spike
Association with bisphosphonates	Prevented by bisphosphonates	Associated with long-term use
Healing	Usually normal	Often delayed

This discovery has led to intense research and debate within the medical community. This phenomenon raised important questions about the long-term effects of medications that alter bone metabolism. While these atypical femoral fractures (AFFs) are rare - especially compared to the common fractures that bisphosphonates prevent - understanding them has become crucial for optimal patient care [14].

This review explains the AFFs caused by long-term bisphosphonate use. We cover how to recognize these fractures, why they occur, what symptoms to look for, and how to diagnose them. We describe treatment options for both incomplete and complete fractures and discuss how to manage patients' bone health afterward. We also emphasize the importance of teaching patients about warning signs and regular monitoring. This review helps doctors use bisphosphonates safely while preventing this rare but serious problem.

## Review

Methods

A comprehensive literature search was conducted using PubMed, Scopus, and Google Scholar databases to identify relevant studies on bisphosphonate-related AFFs. The search was performed in October 2024 and covered publications from January 2005 to October 2024, corresponding to the period when AFFs were first recognized in the literature. The following search terms and combinations were used: "bisphosphonate," "atypical femoral fracture," "atypical femur fracture," "subtrochanteric fracture," "diaphyseal femur fracture," "long-term bisphosphonate use," "bone remodeling suppression," and "osteoporosis treatment complications."

Studies were included if they were published in English-language, peer-reviewed journals and addressed the epidemiology, pathophysiology, clinical presentation, diagnosis, or management of bisphosphonate-related AFFs, including original research articles, systematic reviews, meta-analyses, clinical guidelines, and relevant case series. Studies focusing solely on typical osteoporotic fractures, without mention of atypical fractures, were excluded.

Initial screening was performed by reviewing titles and abstracts to identify potentially relevant articles, followed by full-text assessment of eligible articles. Reference lists of selected articles were manually searched to identify additional relevant studies.

Given the substantial heterogeneity in study designs, outcome measures, and patient populations across the included literature, quantitative meta-analysis was not performed. Instead, data were synthesized narratively across key clinical domains, including epidemiology, pathophysiology, risk factors, clinical presentation, diagnostic approaches, and management strategies. Where available, specific numeric data - including incidence rates, risk ratios, confidence intervals, and p-values from individual studies - were incorporated to provide quantitative context. Consensus findings across multiple studies were emphasized, and when conflicting evidence existed, different perspectives were presented with their respective statistical support.

Defining AFFs

AFFs differ from fractures that occur in typical osteoporosis. Fractures must occur in the femoral shaft. More specifically, these fractures occur from just below the lesser trochanter down to just above where the bone widens near the knee. This location is quite different from typical osteoporotic hip fractures, which occur at the femoral neck or intertrochanteric region near the hip joint. To help physicians consistently identify these fractures, the American Society for Bone and Mineral Research (ASBMR) established specific criteria [15,16], including major and minor features, to guide the diagnosis of AFFs (Table 2).

**Table 2:** ASBMR Diagnostic Criteria for Atypical Femoral Fractures This table is not a copy from the source; it has been generated by the author, Ahmed Mohamed. ASBMR, American Society for Bone and Mineral Research

Major Features (all must be present)	Minor Features (may be present)
Location: between lesser trochanter and supracondylar flare	Prodromal pain in groin or thigh
Minimal or no trauma	Cortical thickening (periosteal/endosteal)
Transverse or short oblique fracture line	Bilateral fractures or symptoms
Originates from lateral cortex	Delayed fracture healing
Complete or incomplete	Comorbidities (vitamin D deficiency, glucocorticoid use)
-	Medial spike (cortical beaking)

Suspecting and identifying AFFs requires clinical and radiological findings. Clinically, patients report minimal or no associated trauma. Fractures occur during routine activities, such as walking, standing, or even just bearing weight on the leg. Some describe a simple misstep or turning motion, whereas a few patients report no identifiable incident. Additionally, a few patients complain of long-standing pain and discomfort in the thigh weeks before the fracture occurs. This stands in stark contrast to typical osteoporotic fractures, which usually occur after falls [17,18].

Radiologically, the fracture line appearance is distinctive. On plain radiographs, the fracture appears transverse or short oblique. Cortical thickening is also pathognomonic, with an associated medial spike - a pointed fragment known as cortical beaking. The fracture line may also show delayed healing or a lack of callus formation after fixation [19,20].

Epidemiology and incidence

Understanding how common AFFs are has been crucial in putting this complication into the proper perspective. The data show that these fractures are rare, particularly when compared to the number of typical osteoporotic fractures that bisphosphonates prevent. Studies have found that the incidence of AFFs ranges from approximately 2-100 cases per 100,000 individuals per year among bisphosphonate users, depending on the population studied and the duration of use [21,22]. These numbers increase with longer treatment durations. Research suggests that the risk is relatively low in the first few years of bisphosphonate use, but it increases significantly after five years, with the highest rates observed in patients who have been on these medications for 8 to 10 years or more [23,24].

One important study from Sweden examined over 12,000 women who had taken bisphosphonates and found that the absolute risk of AFFs was approximately five cases per 10,000 patients per year after five years of use [25]. While these numbers may seem concerning, they must be balanced against the substantial reduction in the typical fractures that these medications prevent [8].

The risk appears to decrease relatively quickly after bisphosphonate discontinuation. Studies have shown that the incidence of AFFs decreases by approximately 70% within two years of discontinuing the medication, although some residual risk may persist for several years, due to the long-lasting effects of bisphosphonates on bone [26].

Individual variations in bone geometry play an important role in determining susceptibility to AFFs. Patients with increased femoral bowing experience even greater mechanical stress on the lateral cortex, where these fractures occur. Certain populations appear to be at an increased risk. Asian populations, particularly those of East Asian descent, show higher rates of AFFs than Caucasian populations. The reasons for this ethnic variation are not entirely clear, but may be related to differences in bone geometry, with Asian individuals often having narrower bones with thicker cortices [27,28].

Pathophysiology: understanding the mechanism

The development of AFFs represents a complex interplay between altered bone biology, mechanical stress, and individual patient factors. Bone is a dynamic tissue that undergoes constant remodeling throughout life. This process involves two main types of cells: osteoclasts, which break down old bone, and osteoblasts, which build new bone. In healthy bone, these processes remain balanced, maintaining bone strength while allowing the skeleton to adapt to changing mechanical demands, and to repair microscopic damage that accumulates with daily activities [29-31].

Osteoporosis develops when this delicate balance is disrupted, and bone resorption outpaces bone formation. This imbalance occurs through multiple mechanisms: osteoclast activity increases, while osteoblast function declines, particularly following estrogen deficiency in postmenopausal women or during normal aging processes. Inflammatory cytokines and hormonal changes further promote osteoclast formation and prolong their lifespan, leading to excessive bone resorption. The result is progressive bone loss, where the trabecular network becomes thinner and more porous, while the cortical bone loses thickness and density. This deterioration of bone mass and microarchitecture increases skeletal fragility and susceptibility to fractures from minimal trauma [32-34].

Bisphosphonates inhibit osteoclasts, leading to a slowing of bone breakdown. Therefore, they shift the balance back toward bone preservation and accumulation [4]. However, bone remodeling serves important purposes beyond maintaining density. It allows the skeleton to repair microdamage, replace old bone with fresh bone, and adapt to mechanical stress patterns. When this remodeling process is suppressed for extended periods, normal repair mechanisms are compromised [35].

Normally, the natural curvature of the femur subjects the lateral cortex of the femoral shaft to high tensile forces during weight-bearing activities, meaning that the bone is pulled apart rather than compressed. These repetitive loading cycles create microscopic fractures and damage in the mechanically stressed region [36-38]. Under normal circumstances, with functioning osteoclasts, these microfractures are continuously identified and repaired, maintaining bone integrity. With bisphosphonates, osteoclast function is severely suppressed for extended periods, compromising the repair mechanism. As a result, microdamage accumulates faster than it can be repaired, particularly in the lateral cortex, where mechanical stress is concentrated. Over time, this accumulated, unrepaired damage makes the region susceptible to stress fracture formation. The periosteal and endosteal thickening observed on radiographs at the fracture site represents the bone's compensatory attempt to reinforce itself in response to accumulating stress and microdamage [39-42].

In addition to suppressing bone remodeling, some researchers have proposed that bisphosphonates may also affect the material properties of bone. Long-term bisphosphonate use might alter the mineral composition and collagen cross-linking in bone, making it more brittle or less able to absorb stress before fracturing [43,44]. These changes in bone quality, combined with the accumulation of unrepaired microdamage, create conditions that favor the development of atypical fractures in mechanically vulnerable areas.

Clinical presentation and warning signs

One of the most important aspects of AFFs is that they often provide warning signs before the bone completely breaks. Many patients report a dull, aching pain in the thigh or groin that develops gradually over weeks or months before the fracture occurs. This pain is located on the outer or front aspect of the thigh and may be described as deep, nagging discomfort. The pain often worsens with weight-bearing activities, such as walking or standing, and may improve with rest. This prodromal pain represents an incomplete fracture or stress reaction of the bone. Unfortunately, these warning signs are sometimes dismissed or attributed to other causes, such as muscle strain, arthritis, or referred pain from the back or hip [22,45,46].

When the fracture is complete, patients experience sudden, severe pain in the thigh, accompanied by an inability to bear weight on the affected leg. Unlike typical falls that may cause hip fractures, fracture events may occur with minimal trauma. Patients describe incidents such as stepping off a curb, pivoting while walking, or simply feeling the leg give way during normal activity.

The bilateral nature of these fractures warrants special mention. When a patient presents with an AFF on one side, a full history and examination must be clearly documented, if there is any contralateral thigh pain, as identifying and treating an incomplete fracture can prevent progression to a complete break [47-50].

Educating first-line doctors, particularly general practitioners and junior emergency medicine doctors, is crucial for the management of AFFs. Many patients on long-term bisphosphonate therapy initially present with vague thigh or groin pain before sustaining a complete fracture. Early recognition of this warning sign is vital, as timely investigation can prevent the progression to displaced fractures. These clinicians should maintain a very low threshold for requesting radiographs of the entire femur, and, when suspicion remains despite negative plain films, further imaging, such as magnetic resonance imaging (MRI) or bone scan, should be considered. Improving awareness at the primary and emergency care levels ensures earlier diagnosis, reduces morbidity, and allows for preventive strategies, such as prophylactic fixation or modification of osteoporosis treatment [51].

Educating patients about AFFs is a critical component of safe bisphosphonate therapy. At treatment initiation and during follow-up visits, patients should be informed about the benefits of bisphosphonates in preventing typical osteoporotic fractures and the risk of AFFs with their prolonged use. Patients should be instructed to report any new or unusual pain in the thigh, groin, or hip region, even if the pain seems minor or unrelated to the injury [52].

Diagnostic approaches

Accurate and timely diagnosis of AFFs requires a combination of clinical suspicion, appropriate imaging, and knowledge of characteristic features. Plain radiography remains the first-line imaging modality. Standard anteroposterior and lateral views of the femur should be obtained if an AFF is suspected. These images can reveal the characteristic transverse or short oblique fracture line originating from the lateral cortex, medial spike (if present), and cortical thickening.

When X-rays are negative or equivocal, but clinical suspicion remains high, advanced imaging is necessary. MRI has excellent sensitivity for detecting incomplete AFFs and stress reactions in bone. MRI can show bone marrow edema, periosteal reaction, and subtle cortical breaks that might not be visible on plain films. This makes MRI particularly valuable for evaluating patients with prodromal thigh pain and negative radiographic findings [53].

Bone scans play a role in the early detection of AFFs in patients taking bisphosphonates. They are highly sensitive in identifying areas of increased bone turnover or stress along the femoral shaft, indicating incomplete or impending fractures. However, bone scans are not specific and may show uptake in other conditions, such as metastases or infection; therefore, abnormal findings should be confirmed with X-rays or MRI, which provide a detailed assessment of cortical integrity and fracture completeness. Bone scans are also useful for evaluating both femurs, as atypical fractures often occur bilaterally [54].

An important diagnostic consideration is the evaluation of the contralateral femur when an AFF is identified on one side. Current recommendations suggest obtaining radiographs of the opposite leg in all patients diagnosed with AFF, even if the leg is asymptomatic. If the patient reports pain in the opposite thigh, MRI should be strongly considered to detect incomplete fractures that may not yet be visible on radiographs [55,56].

Management of incomplete fractures

The discovery of an incomplete AFF presents both challenges and opportunities. The first and most crucial step is the discontinuation of bisphosphonate therapy. Stopping the medication allows bone remodeling to gradually resume, which should permit the body’s natural repair mechanisms to address stress fractures [57,58].

Activity modification is another key component of management. Patients with incomplete AFF should reduce weight-bearing activities in the affected leg. Many clinicians recommend using crutches or a walker to minimize stress on the bone during healing. The duration of restricted weight-bearing lasts for at least several weeks to a few months. Nutritional optimization is important, and ensuring adequate calcium and vitamin D intake supports bone healing and helps restore normal bone metabolism [59,60].

Close monitoring is essential for conservative management. Follow-up radiographs should be obtained regularly, every six to eight weeks initially, to assess whether the fracture is healing, remaining stable, or progressing. The decision between conservative management and prophylactic surgery depends on several factors. Small, incomplete fractures with minimal cortical involvement and no significant symptoms can often be managed conservatively. However, fractures that involve more than half of the cortical width, those that show progression on follow-up imaging, or those that cause significant symptoms, despite conservative measures, may warrant prophylactic surgical stabilization [61,62].

Management of complete fractures

When the AFF is complete, surgical intervention is the only treatment option. Intramedullary nailing is the preferred surgical technique for most complete AFFs. This procedure involves inserting a metal rod through the center of the femur, which provides support along the length of the bone. The rod is locked in place with screws, above and below the fracture site [63].

The surgical approach must be carefully planned, because AFFs present unique technical challenges that distinguish them from typical osteoporotic fractures. The transverse fracture pattern, rather than the oblique or spiral configuration seen in typical fractures, provides less stability and fewer interlocking surfaces for reduction. This makes it more difficult to achieve and maintain proper anatomical alignment during surgery. Additionally, the cortical thickening and sclerosis that characterize these fractures create a hard, dense bone that is difficult to work with. Surgeons often encounter difficulties when attempting to pass guidewires, reamers, or screws, through abnormally thickened cortices. Hardened bone may resist standard drilling techniques. The brittle nature of bone, resulting from prolonged suppression of remodeling and altered material properties, can cause additional fracture lines to propagate during reduction maneuvers or hardware insertion. Surgeons must be careful to avoid iatrogenic fracture extension, particularly at the proximal and distal ends of the nail, where stress concentrations are observed. Additionally, the nail length should extend beyond any cortical beaking, as stopping short may leave a stress riser that could propagate future fractures [64,65].

Furthermore, the impaired healing capacity of the bone necessitates a meticulous surgical technique to optimize the mechanical environment for fracture union. Achieving compression at the fracture site may be more difficult than with typical fractures, and the surgeon must balance adequate stability with preservation of the periosteal blood supply, which becomes even more critical when bone remodeling is compromised. These technical considerations often result in longer operative times and require experienced surgical teams familiar with the unique characteristics of AFFs [12,66].

Postoperative and long-term management

After surgical fixation of AFFs, clinicians and patients face important decisions regarding long-term management. Careful monitoring of healing is essential, as these fractures are prone to delayed union or nonunion due to suppressed bone turnover from long-term bisphosphonate use. Healing often takes 6-12 months or longer; therefore, regular radiographic follow-up and surveillance for implant failure are required. Weight-bearing is usually restricted initially and advanced gradually as healing progresses [67].

Bisphosphonates should be discontinued, and a drug holiday is recommended, usually for at least 1 to 10 years, with many patients requiring longer breaks of up to two to five years, depending on bone health and fracture risk. The residual effects of bisphosphonates persist for years after discontinuation, as they remain bound to the bone and are released slowly, meaning that bone mineral density often remains stable during this period. However, this creates a therapeutic dilemma: continuing therapy increases the risk of another AFF, whereas stopping therapy may raise the risk of typical osteoporotic fractures [68].

During the drug holiday, bone health should be optimized with calcium and vitamin D supplementation, and monitoring with bone density scans and bone turnover markers is recommended. Anabolic agents, such as teriparatide and abaloparatide, represent one alternative. These medications stimulate bone formation and increase bone density through a mechanism that is completely different from that of anti-resorptive drugs, actively building new bone rather than simply preventing bone loss. Teriparatide is a recombinant form of parathyroid hormone that stimulates osteoblast activity and promotes new bone formation when administered intermittently. Abaloparatide works through a similar mechanism, selectively activating parathyroid hormone receptor pathways that favor bone building over bone resorption, and may be appropriate for patients with a high fracture risk who have experienced AFFs [69].

Importantly, during the follow-up period, special consideration should be directed to asking patients about any contralateral thigh pain. These fractures can be bilateral, occurring simultaneously or at different times. Some studies suggest that up to 20%-30% of patients with AFFs on one side will develop them on the other side within a year or two if preventive measures are not taken [48,70].

## Conclusions

AFFs represent a rare but significant complication of long-term bisphosphonate therapy, occurring when prolonged suppression of bone remodeling leads to the accumulation of unrepaired microdamage in the mechanically stressed femoral shaft. Although the incidence increases significantly after five years of use, particularly in high-risk populations such as patients of Asian descent or those on glucocorticoids, the overall risk remains low compared to the number of typical osteoporotic fractures that bisphosphonates prevent. Early recognition of prodromal symptoms, prompt imaging evaluation, and timely intervention can prevent the progression from incomplete to complete fractures. Management requires immediate discontinuation of bisphosphonates, with treatment ranging from conservative measures for incomplete fractures to surgical fixation for complete fractures.

Long-term care involves careful reassessment of osteoporosis treatment needs, with options including drug holidays for lower-risk patients or a transition to alternative medications, such as anabolic agents, for those requiring continued therapy. The key to optimal outcomes lies in clinical vigilance, patient education about warning symptoms, regular monitoring of patients on long-term therapy, and individualized decision-making that balances the substantial fracture-prevention benefits of bisphosphonates against the small but real risk of AFFs. By understanding these fractures and managing bisphosphonate therapy thoughtfully, clinicians can continue to provide excellent osteoporosis care while minimizing the risk of this rare complication.
